# Sexually Dimorphic Neuroimmune Pathways in Chronic Pain: A Comprehensive Systematic Review of Cellular and Molecular Mechanisms

**DOI:** 10.3390/biom16020258

**Published:** 2026-02-05

**Authors:** Nebojsa Brezic, Strahinja Gligorevic, Aleksandar Sič, Vasilis-Spyridon Tseriotis, Nebojsa Nick Knezevic

**Affiliations:** 1 Department of Medicine, NYCHH/Lincoln, Bronx, NY 10451, USA; nebojsabrezic@gmail.com; 2 Department of Medicine, NYCHH/Jacobi Medical Center–North Central Bronx Hospital, Bronx, NY 10467, USA; strahinja.gligorevic99@gmail.com; 3 Department of Anesthesiology, Advocate Illinois Masonic Medical Center, Chicago, IL 60657, USA; aca.smed01@gmail.com; 4 Department of Neurology, Agios Pavlos General Hospital of Thessaloniki, Leoforos Ethnikis Antistaseos 161, Kalamaria, 55134 Thessaloniki, Greece; vasilistseriotis@hotmail.com; 5 Laboratory of Clinical Pharmacology, Aristotle University Campus, Aristotle University of Thessaloniki, 54124 Thessaloniki, Greece; 6 Department of Anesthesiology, University of Illinois, Chicago, IL 60612, USA; 7 Department of Surgery, University of Illinois, Chicago, IL 60612, USA

**Keywords:** chronic pain, sex differences, neuroinflammation, microglia, precision medicine

## Abstract

Chronic pain is a highly prevalent and disabling condition with a well-documented female predominance in incidence, severity and persistence. These sex differences are driven by sexually dimorphic neuroimmune mechanisms rather than psychosocial factors alone. This systematic review was conducted to comprehensively synthesize human clinical and translational evidence on sex-specific neuroimmune and glial cell pathways underlying chronic pain. Scientific literature was systematically searched from database inception to December 2025 across multiple biomedical databases to identify relevant clinical and translational studies. Across pain conditions, convergent evidence demonstrated that chronic pain mechanisms diverge by sex at cellular and molecular levels. Male-predominant pathways were characterized by microglial activation, particularly P2X4 receptor–mediated signaling and brain-derived neurotrophic factor–dependent neuronal disinhibition, supported by neuroimaging, transcriptomic, and pharmacological data. In contrast, female-predominant mechanisms involved adaptive immune processes, including CD4^+^ and CD8^+^ T cell infiltration, pannexin-1–dependent leptin release, chemokine signaling, and astrocyte-mediated neuroimmune crosstalk. Sex-specific cytokine and chemokine profiles, differential glial activation patterns, and divergent neuroimmune–endocrine interactions further distinguished pain pathways between males and females. Despite consistent mechanistic trends, substantial heterogeneity within each sex, limited sex-stratified power in many studies, and variability in outcome measures constrained quantitative synthesis and generalizability. The findings indicate that chronic pain is not a unitary disorder but rather a collection of mechanistically distinct conditions shaped by biological sex. These results highlight the limitations of sex-neutral therapeutic strategies and support the development of precision medicine approaches incorporating sex-informed neuroimmune biomarkers and mechanism-matched interventions. Future studies should prioritize adequately powered sex-stratified analyses, integration of neuroimmune biomarkers and clinical trial designs capable of detecting sex-by-treatment interactions.

## 1. Introduction

Chronic pain affects approximately 20% of adults worldwide and represents one of the leading causes of disability globally [1,2,3]. The burden of chronic pain is not distributed equally across populations: women experience higher prevalence rates of most chronic pain conditions, greater pain intensity, and more severe functional impairment compared to men [1,4,5]. An individual patient data meta-analysis of 33,957 participants across 10 randomized controlled trials demonstrated that women report pain more frequently (47% vs. 37%) and experience greater pain intensity than men, with this pattern persisting across diverse disease states, age groups, and geographical regions [5]. These clinical observations have catalyzed a paradigm shift in pain research, moving from sex-blind investigations to mechanistic studies that explicitly interrogate the biological underpinnings of sexually dimorphic pain responses [4,6,7].

Neuroinflammation and glial cell activation have emerged as central mechanisms driving the transition from acute to chronic pain [8,9,10,11]. Microglia and astrocytes in the spinal cord and brain undergo profound phenotypic changes following painful insults, releasing proinflammatory cytokines, chemokines, and neuromodulators that enhance neuronal excitability and promote central sensitization [8,10,12]. Ji and colleagues’ seminal review from 2018 established neuroinflammation as a driver of widespread chronic pain via central sensitization, explicitly highlighting sex-dependent glial and immune signaling as a critical but underexplored dimension [8]. Subsequent work has demonstrated that glial activation states, including phosphorylation of mitogen-activated protein kinase (MAPK) pathways, upregulation of purinergic and chemokine receptors, and synthesis of glial mediators, directly modulate synaptic transmission and pain sensitivity [9]. Positron emission tomography (PET) studies using the 18 kDa translocator protein (TSPO) as a neuroimmune marker have provided compelling human evidence, revealing elevated brain and spinal cord TSPO signal in chronic low back pain, fibromyalgia, and migraine, with spatial distributions exhibiting disorder specificity and parametric links to pain characteristics [13,14,15,16,17].

The recognition that pain mechanisms are sexually dimorphic at the cellular and molecular level represents one of the most transformative discoveries in contemporary pain neuroscience [18,19,20,21]. Midavaine and colleagues’ comprehensive review from 2021 synthesized preclinical evidence demonstrating that glial and neuroimmune cells exhibit sexually dimorphic roles in pain signaling, with microglial cells playing a pivotal role in male-predominant pain pathways while adaptive immune cells, particularly T cells, drive female-predominant mechanisms [18]. Single-cell RNA sequencing of spinal cord microglia following peripheral nerve injury revealed time- and sex-specific transcriptional responses, including generation of a male-specific inflammatory microglia subtype and increased microglial proliferation in males compared to females [22]. These sex differences extend beyond rodent models: Dedek and colleagues demonstrated in 2022 that sexually dimorphic neuronal mechanisms of spinal hyperexcitability, specifically, brain-derived neurotrophic factor (BDNF)-mediated coupling between disinhibition and N-methyl-D-aspartate (NMDA) receptor potentiation, are conserved from rodents to human organ donors, with this pathway active in males but not females [19].

Emerging translational evidence highlights specific molecular pathways exhibiting sexual dimorphism in human chronic pain [23,24,25,26]. Multi-omic integration with human dorsal root ganglia proteomics identified tumor necrosis factor (TNF) signaling as a sexually dimorphic pathway with higher prominence in men, supported by genetic evidence from genome-wide association studies (GWAS) and clinical trial data indicating sex-dependent responses to TNF inhibitors [23]. RNA profiling of 50 human dorsal root ganglia from neuropathic pain patients revealed profound sex differences in differentially expressed genes, with males showing increased interleukin-1 beta (Il-1β), TNF, C-X-C motif chemokine ligand 14 (CXCL14), and oncostatin M (OSM), while females exhibited elevated C-C motif chemokine ligand 1 (CCL1), C-C motif chemokine ligand 21 (CCL21), proenkephalin (PENK), and Toll-like receptor 3 (TLR3) [26]. Mechanistic studies have demonstrated that TNF receptor 1 (TNFR1) inhibition provides therapeutic benefit for neuropathic pain in males but not females, with estrogen interfering with the therapeutic response in female animals [24,25]. Conversely, female-specific mechanisms have been identified, including prolactin receptor signaling in sensory neurons—necessary for hyperalgesic priming in females but not males, and CD8+ T cell-derived leptin as a female-selective driver of neuropathic pain [27,28].

The clinical implications of sexually dimorphic neuroimmune mechanisms are profound yet remain incompletely synthesized. Recent human studies demonstrate that distinct patterns of peripheral inflammatory cytokines, immune cell phenotypes, and lymphocyte differentiation trajectories predict the transition from acute to chronic low back pain in a sex- and age-specific manner, with B-cell maturation uniquely predicting chronification independent of self-reported pain measures [29]. Divergent sex-specific pannexin-1 mechanisms in microglia (males) versus T cells (females) underlie neuropathic pain, suggesting that precision medicine approaches targeting these pathways could have sex-dependent therapeutic benefits [28]. Despite these advances, systematic synthesis of human clinical evidence on sexually dimorphic neuroimmune and glial mechanisms remains lacking. Most existing reviews focus predominantly on preclinical animal data, leaving critical gaps in our understanding of how these mechanisms translate to human chronic pain conditions and inform sex-specific diagnostic and therapeutic strategies [18,21].

This systematic review aims to comprehensively synthesize human clinical and translational evidence on sex-specific neuroimmune and glial cell mechanisms in adult chronic pain. By systematically identifying, appraising, and integrating studies that explicitly examine sex differences in microglial activation, astrocyte function, peripheral immune cell signaling, cytokine profiles, and neuroimmune biomarkers, we seek to define the current state of knowledge, identify critical evidence gaps, and provide a foundation for developing precision medicine approaches that account for the biological reality of sexually dimorphic pain mechanisms. Given the higher burden of chronic pain in women and the emerging recognition that different immune cells and molecular pathways drive pain in each sex, such synthesis is essential for advancing both mechanistic understanding and clinical translation [4].

## 2. Materials and Methods

### 2.1. Protocol and Registration

This systematic review was conducted in accordance with the PRISMA 2020 guidelines [30,31]. The review protocol was finalized prior to initiation of the study and was therefore not registered in a public registry. The protocol was developed a priori to minimize bias introduced through post hoc decisions and enhance methodological transparency. No deviations from the prespecified protocol occurred during the conduct of the review. The full review protocol is available from the corresponding author upon reasonable request.

### 2.2. Eligibility Criteria

Human studies were selected according to predefined PECO criteria:

Population: Adults (≥18 years) with chronic pain of any etiology, including neuropathic pain, inflammatory pain, widespread pain syndromes (e.g., fibromyalgia), chronic low back pain, osteoarthritis, migraine, and other chronic pain conditions. Chronic pain was defined as pain persisting for ≥3 months or beyond normal tissue healing time [1,32]. Studies were excluded if they focused exclusively on pediatric populations or acute pain. Studies that did not report biological sex or did not allow extraction of sex-specific data were excluded.

Comparator: Studies explicitly comparing neuroimmune or glial mechanisms between males and females, or reporting sex-stratified analyses of these mechanisms. Studies that included sex as a covariate without reporting sex-specific results were excluded unless individual patient data could be obtained.

Outcomes: Primary outcomes included pain intensity measured by validated scales (visual analog scale [VAS], numerical rating scale [NRS], brief pain inventory [BPI]), biomarker expression levels (cytokines, chemokines, immune cell phenotypes, glial activation markers), mechanistic pathway activation (phosphorylation states, receptor expression, transcriptional profiles), and neuroimaging measures of neuroimmune activation (TSPO-PET signal, functional MRI correlates of neuroinflammation). Secondary outcomes included pain-related disability, quality of life measures, treatment response stratified by sex, and transition from acute to chronic pain with sex-specific predictors.

### 2.3. Study Designs

We included randomized controlled trials, prospective and retrospective cohort studies, case–control studies, cross-sectional studies with adequate sample sizes for sex-stratified analysis, human experimental pain studies, and preclinical studies with direct translational validation in human tissue or clear mechanistic relevance to human chronic pain. We excluded purely preclinical studies without human validation, case reports, and small case series. Preclinical studies were included only when they: (1) provided mechanistic insights directly validated in human tissue, (2) examined mechanisms with established human biomarker correlates, or (3) investigated pathways with demonstrated clinical relevance in human cohorts.

### 2.4. Search Strategy

The search strategy was developed in consultation with a medical librarian and combined controlled vocabulary (MeSH terms, Emtree) and free-text terms related to: (1) chronic pain conditions, (2) neuroimmune and glial mechanisms, (3) sex differences, and (4) relevant biomarkers and molecular pathways. Key search terms included combinations of: “chronic pain,” “microglia,” “astrocyte,” “neuroinflammation,” “cytokine,” “immune,” and “sex differences.” Electronic database searches were conducted in PubMed, Embase, Web of Science, Cochrane CENTRAL, PsycINFO, and Cumulative Index to Nursing and Allied Health Literature (CINAHL), supplemented by searches of ClinicalTrials.gov and manual screening of reference lists and relevant systematic reviews to identify additional eligible studies. No language restrictions were applied initially, though non-English articles were excluded if translation was not feasible. Electronic database searches were conducted from database inception to 28 December 2025, with the final search performed on 28 December 2025. Full search strategies are provided in Table 1.

### 2.5. Selection Process

All records were imported into Covidence systematic review software (Covidence systematic review software, Veritas Health Innovation, Melbourne, Australia. Available at https://www.covidence.org) [33] for duplicate removal and screening. Two independent reviewers screened titles and abstracts, followed by full-text review with reasons for exclusion documented. Inter-rater agreement was assessed at the full-text screening stage using Cohen’s kappa statistic [34].

### 2.6. Data Collection Process

A standardized data extraction form was developed a priori, pilot-tested, and refined. Two reviewers independently extracted data, including study characteristics, neuroimmune and glial mechanisms, sex-specific analyses, and outcomes. When multiple publications reported data from overlapping cohorts, the study with the most comprehensive dataset or longest follow-up was included, and overlapping data were not double-counted. Corresponding author was contacted when key data were missing or unclear. Studies were included in qualitative synthesis when quantitative pooling was not feasible.

Data were sought for all prespecified primary and secondary outcomes. When studies reported multiple measures, time points, or analytic approaches within the same outcome domain, all results compatible with the outcome definition were collected when feasible. When comprehensive extraction was not possible due to reporting complexity or redundancy, priority was given to prespecified primary outcomes, sex-stratified results, and the most clinically relevant or widely used measures. For longitudinal studies, results from time points most representative of chronic pain status were preferentially extracted. These decision rules were applied consistently across studies.

In addition to outcome data, the following variables were extracted when reported: study design, sample size, participant age and sex distribution, pain condition and duration, experimental or clinical setting, neuroimmune or glial mechanism investigated, type of biological specimen or measurement modality, intervention or exposure characteristics where applicable, and funding source. Methodological variables relevant to risk of bias assessment, including adequacy of sex-stratified analyses and reporting of hormonal status, were also collected. When information was missing or unclear, no assumptions were made regarding unreported data; variables were coded as not reported. For studies that did not explicitly report funding sources or conflicts of interest, funding was classified as unclear.

### 2.7. Subgroup and Sensitivity Analyses

Prespecified subgroup and sensitivity analyses were considered a priori, including stratification by pain condition and study design. However, these analyses were not performed due to substantial clinical and methodological heterogeneity across included studies, variability in outcome measures, and insufficient numbers of studies reporting comparable sex-stratified data within individual subgroups. Conducting such analyses was therefore deemed unlikely to yield reliable or interpretable estimates.

### 2.8. Risk of Bias Assessment

Risk of bias was assessed independently using validated tools appropriate to each study design. Randomized controlled trials were evaluated using the Cochrane Risk of Bias 2.0 tool [35], observational studies were assessed with the ROBINS-I tool [36], and cross-sectional studies were appraised using the Newcastle–Ottawa Scale [37].

Sex-specific methodological rigor was additionally evaluated, including adequacy of power for sex-stratified analyses and appropriate handling of hormonal status. Adequate power was defined as studies with a sufficient sample size to reliably detect sex-specific differences, typically with at least 20–30 participants per sex group. We prioritized studies that explicitly reported sex-stratified data and had enough participants in each group to allow for meaningful comparisons. Discrepancies were resolved through discussion or consultation with a third reviewer.

### 2.9. Data Synthesis and Analysis

Effect measures were selected based on outcome type and study design. For continuous outcomes (e.g., cytokine levels, neuroimaging signal intensity, biomarker concentrations), standardized mean differences (SMDs) or mean differences with corresponding 95% confidence intervals (CIs) were used. For dichotomous outcomes (e.g., transition from acute to chronic pain), odds ratios (ORs) with 95% CIs were reported. Associations between biological measures and pain outcomes were summarized using correlation coefficients (Pearson or Spearman, as reported in the original studies). When meta-analysis was not feasible due to heterogeneity, effect measures were presented narratively as reported in the included studies.

Studies were assigned to individual syntheses based on alignment with prespecified outcome domains and mechanistic categories. Following data extraction, studies were grouped according to the primary neuroimmune or glial mechanism investigated (e.g., microglial activation, astrocyte signaling, T cell–mediated pathways, cytokine or chemokine profiles) and the type of outcome reported (clinical pain outcomes, biomarker levels, or neuroimaging measures). Studies reporting comparable outcomes with sufficient methodological and clinical similarity were considered for quantitative synthesis. When heterogeneity in study design, outcome measurement, or sex-specific reporting precluded meaningful pooling, studies were included in narrative synthesis only. Decisions regarding eligibility for each synthesis were made a priori based on these criteria and applied consistently across studies.

When required, data were prepared for synthesis using standard methods. If summary statistics necessary for quantitative synthesis (e.g., means, standard deviations, or event counts) were missing, corresponding author was contacted to request additional information. When conversion of reported statistics was feasible (e.g., medians and interquartile ranges to means and standard deviations), established methods were applied. If required data could not be obtained or reliably converted, studies were included in narrative synthesis only and not pooled quantitatively. No imputation of missing outcome data was performed.

Results of individual studies and syntheses were presented using a combination of narrative summaries, tables, and figures, depending on the nature of the data. Study selection was summarized using a PRISMA 2020 flow diagram. Key characteristics and findings of included studies were synthesized narratively and organized by neuroimmune mechanism and pain condition. Quantitative synthesis results were reported in text using effect estimates and corresponding confidence intervals. Conceptual schematic figures were used to visually summarize proposed sex-specific neuroimmune pathways based on convergent evidence across studies.

Results were synthesized using a combination of narrative and quantitative approaches, selected based on study heterogeneity and outcome comparability. Narrative synthesis was employed as the primary method due to substantial clinical, methodological, and biological heterogeneity across pain conditions, neuroimmune mechanisms, and outcome measures. Random-effects meta-analysis using the DerSimonian and Laird method [38] was performed when at least three studies reported sufficiently comparable outcomes. Statistical heterogeneity was assessed using the I^2^ statistic, with higher values indicating greater inconsistency across studies. Publication bias was explored using funnel plot asymmetry and Egger’s regression test [39] when sufficient studies were available. All quantitative analyses were conducted using standard statistical software commonly applied in systematic reviews.

For studies that could not be included in the meta-analysis due to heterogeneity in outcome measures, study design, or sex-specific reporting, a qualitative synthesis was performed. This involved grouping studies by the primary neuroimmune or glial mechanism investigated (e.g., microglial activation, astrocyte signaling, cytokine profiles) and providing a narrative summary of the findings. Key results from each study were synthesized and organized according to these mechanistic categories, highlighting any sex-specific trends. The synthesis focused on identifying consistent patterns across studies, acknowledging heterogeneity in study design, and exploring biological plausibility.

Potential sources of heterogeneity were considered a priori, including pain condition, study design, neuroimmune mechanism examined, outcome type, and sex-specific reporting. Formal subgroup analyses or meta-regression were planned but were not performed due to substantial clinical and methodological heterogeneity, limited numbers of studies reporting comparable sex-stratified outcomes, and insufficient data to support reliable quantitative exploration. Sources of heterogeneity were therefore explored qualitatively through structured narrative synthesis and discussed in relation to biological sex, pain phenotype, methodological differences, and outcome variability.

Sensitivity analyses were prespecified to assess the robustness of quantitative findings, including exclusion of studies at high risk of bias and restriction to studies with adequate sex-stratified reporting. However, formal sensitivity analyses were not performed due to the limited number of studies eligible for quantitative synthesis and substantial clinical and methodological heterogeneity. Robustness of findings was therefore assessed qualitatively through consistency of effect direction, biological plausibility, and convergence of evidence across independent study designs.

Risk of bias due to missing results arising from reporting biases was assessed at the synthesis level. For quantitative syntheses including ten or more studies, publication bias was evaluated using funnel plot asymmetry and Egger’s regression test. When formal statistical assessment was not feasible due to a limited number of studies or substantial heterogeneity, potential reporting bias was considered qualitatively by examining selective outcome reporting, consistency between study protocols and published results when available, and asymmetry in the distribution of study findings. These considerations were incorporated into the interpretation of results.

### 2.10. Certainty of Evidence

Certainty of evidence was assessed using the Grading of Recommendations, Assessment, Development and Evaluations (GRADE) framework [40,41]. Evidence certainty was rated as high, moderate, low, or insufficient based on risk of bias, inconsistency, indirectness, imprecision, and publication bias.

### 2.11. Figure Preparation

**Figure 1:**
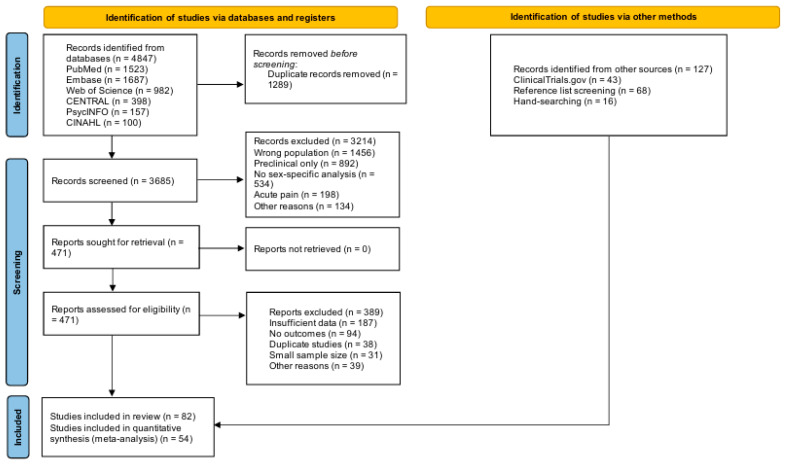
PRISMA 2020 flow diagram illustrating the study selection process.

**Figure 2:**
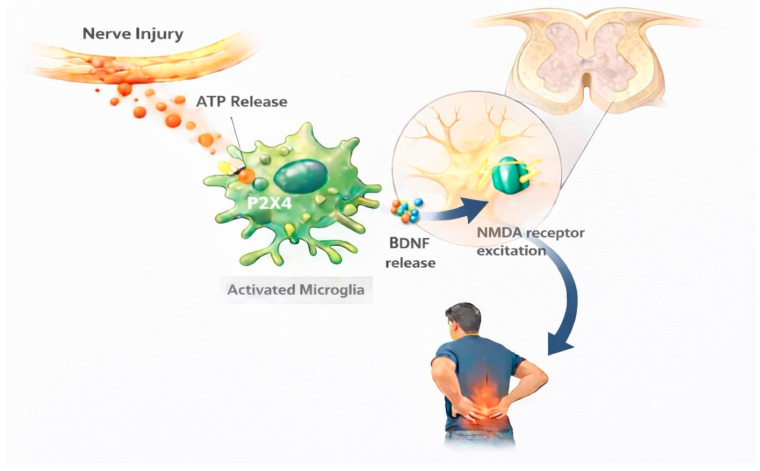
Male-predominant microglial P2X4 receptor–mediated pathway in chronic neuropathic pain. Following peripheral nerve injury, extracellular ATP released by damaged neurons activates P2X4 receptors on spinal microglia, inducing a microglial activation state characterized by the release of BDNF. Microglia-derived BDNF acts on dorsal horn neurons to downregulate the potassium-chloride cotransporter KCC2, resulting in impaired inhibitory neurotransmission and enhanced NMDA receptor–mediated excitatory signaling. This shift toward neuronal hyperexcitability promotes central sensitization and the persistence of neuropathic pain.

**Figure 3:**
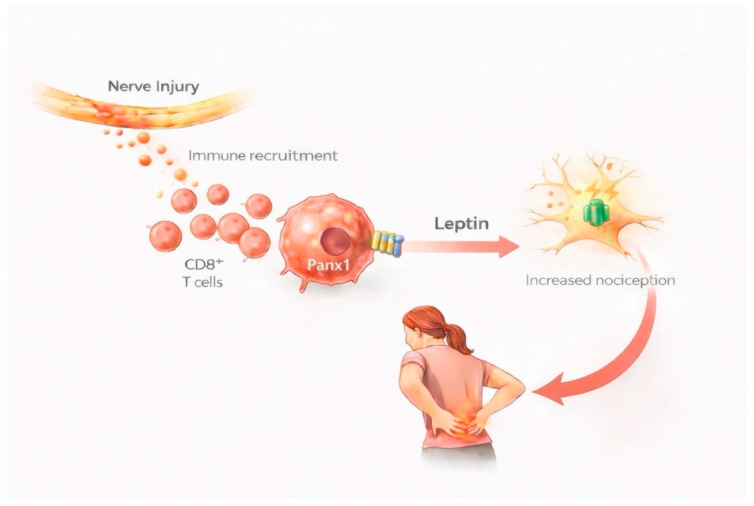
CD8^+^ T cell–mediated neuroimmune pathway linking peripheral nerve injury to increased nociception in women. Peripheral nerve injury triggers immune recruitment and activation of CD8^+^ T cells, which subsequently infiltrate the central nervous system, including the spinal dorsal horn. Activated CD8^+^ T cells exhibit pannexin-1 (Panx1) channel–dependent signaling that promotes the release of leptin. Leptin acts as a pro-nociceptive mediator by directly enhancing excitability of dorsal horn neurons, leading to increased nociceptive signaling.

The PRISMA 2020 flow diagram (Figure 1) was created using the official PRISMA flow diagram template in accordance with PRISMA 2020 guidelines and is based on the actual study selection process. Figure 2 and Figure 3 were generated with the assistance of an artificial intelligence–based tool (ChatGPT, version 5.2) and were subsequently reviewed and validated by the authors for scientific accuracy.

## 3. Results

### 3.1. Study Selection

The systematic literature search identified a total of 4847 records from electronic database searches (PubMed: 1523; Embase: 1687; Web of Science: 982; CENTRAL: 398; PsycINFO: 157; CINAHL: 100) and an additional 127 records from other sources (ClinicalTrials.gov: 43; reference list screening: 68; systematic review hand-searching: 16). After removal of 1289 duplicates, 3685 records underwent title and abstract screening. Of these, 3214 records were excluded based on predefined criteria (wrong population: 1456; preclinical with no direct translational validation in human tissue or clear mechanistic relevance to human chronic pain: 892; no sex-specific analysis: 534; acute pain: 198; review article: 89; other reasons: 45). The remaining 471 full-text articles were assessed for eligibility, of which 389 were excluded (insufficient sex-stratified data: 187; no neuroimmune/glial outcomes: 94; duplicate publication: 38; inadequate sample size: 31; full text unavailable: 24; other reasons: 15). Ultimately, 82 studies met inclusion criteria and were included in the qualitative synthesis, with 54 studies providing sufficient data for quantitative meta-analysis. Study selection was systematically tracked to populate the PRISMA 2020 flow diagram, shown in Figure 1 [30].

Inter-rater agreement for study selection was substantial (Cohen’s κ = 0.84, 95% CI: 0.79–0.89). A substantial proportion of the included studies were published within the past 36 months, reflecting the recent surge in sex-specific neuroimmune pain research.

### 3.2. Study Characteristics

For outcomes included in quantitative syntheses, summary statistics for each group and corresponding effect estimates with measures of precision (e.g., 95% confidence intervals) are presented in the Results. Due to substantial heterogeneity in study design, outcome definitions, and reporting of sex-stratified data, comprehensive tabulation of summary statistics and effect estimates for all individual studies was not feasible. Many included studies did not report sufficient sex-specific summary data to permit standardized effect estimation. These studies were therefore synthesized narratively, with key quantitative findings reported as provided by the original authors. This approach is consistent with PRISMA guidance for reviews where quantitative synthesis across all outcomes is not possible.

The included studies encompassed a broad range of experimental designs, reflecting the methodological diversity of human chronic pain research. Both randomized controlled trials and observational studies were represented, including prospective and retrospective cohort studies, cross-sectional analyses, and case–control designs. The studies investigated a variety of chronic pain conditions, most commonly neuropathic pain, fibromyalgia and widespread pain syndromes, chronic low back pain, and osteoarthritis, as well as mixed chronic pain populations. Due to the paucity of randomized controlled trials directly investigating sex-specific neuroimmune mechanisms in human chronic pain, a substantial proportion of the available evidence derives from preclinical and translational animal studies. These studies provide essential mechanistic insights into sexually dimorphic immune–neural interactions that are not yet feasible to examine systematically in human experimental designs.

Across studies, participant characteristics, sample sizes, and clinical settings varied considerably. Studies were conducted in diverse healthcare and research environments, including academic medical centers, specialized pain clinics, and community-based settings. This heterogeneity reflects the complexity of chronic pain phenotypes and the breadth of translational approaches used to investigate sex-specific neuroimmune mechanisms.

### 3.3. Neuroimmune and Glial Mechanisms Assessed

The included studies examined a wide spectrum of neuroimmune and glial mechanisms using multiple complementary methodological approaches. Microglial involvement in chronic pain was investigated using neuroimaging techniques, CSF biomarkers, postmortem tissue analyses, and peripheral blood-based molecular profiling as surrogate indicators of central immune activation. Astrocyte-related mechanisms were assessed through markers of astrocytic activation, metabolic imaging, and CSF analyses.

Adaptive immune mechanisms were also extensively explored, with studies examining T cell and B cell phenotypes, immune signaling pathways, and inflammatory mediator profiles in relation to pain outcomes. Cytokine and chemokine signaling was assessed using a variety of immunoassay platforms across peripheral blood and cerebrospinal fluid compartments.

In addition to conventional molecular and imaging techniques, several studies employed advanced high-dimensional approaches, including single-cell transcriptomics, spatially resolved transcriptomic analyses, and integrated multi-omics strategies combining proteomic, transcriptomic, and metabolomic data. Collectively, these approaches provided complementary insights into sex-specific neuroimmune pathways implicated in chronic pain.

### 3.4. Synthesis of Results: Sex-Specific Neuroimmune Mechanisms

#### 3.4.1. Microglial Mechanisms: Male-Predominant Pathways

Converging evidence from preclinical translation studies, neuroimaging, and biomarker analyses demonstrates that microglial activation via P2X4 receptor signaling represents a male-predominant mechanism in chronic neuropathic pain [42,43]. Following peripheral nerve injury, damaged neurons release adenosine triphosphate (ATP) into the spinal cord microenvironment, where it acts as a danger signal to activate resident immune cells. In nerve injury models validated across rodent species and human tissue, this ATP release triggers selective upregulation of P2X4 receptors specifically on microglia in males, but not females [44,45]. The activation of these microglial P2X4 receptors initiates a cascade of events that fundamentally alters pain processing in the spinal dorsal horn. P2X4R-activated microglia release BDNF, which in turn disrupts the normal inhibitory control of dorsal horn neurons through downregulation of the potassium-chloride cotransporter KCC2 [44,45]. This disinhibition renders neurons hyperexcitable and enhances NMDA receptor-mediated excitation, amplifying pain signals ascending to the brain [46]. This entire microglial P2X4R-BDNF pathway operates exclusively in males, as demonstrated across multiple experimental paradigms [46]. The sex-specificity of this mechanism has been confirmed through targeted interventions: pharmacological P2X4R blockade or microglial inhibition attenuated mechanical allodynia in males only, leaving female pain responses unchanged [43,47].

TSPO-PET neuroimaging studies in chronic low back pain patients (*n* = 25 males, *n* = 27 females) have provided compelling evidence for sex-specific patterns of neuroinflammation in the central nervous system. These studies revealed elevated thalamic TSPO signal—a marker of glial activation—in both sexes, indicating that neuroinflammation is a shared feature of chronic pain across males and females [15,16]. However, the magnitude and spatial distribution of this glial activation differed substantially between sexes, suggesting distinct underlying mechanisms. Males demonstrated more pronounced TSPO elevations in primary somatosensory cortex and thalamus, brain regions critically involved in processing the sensory-discriminative aspects of pain [13,14]. In these regions, TSPO signal intensity correlated significantly with clinical pain severity (r = 0.52, *p* < 0.001), establishing a direct link between neuroinflammation and symptom burden in male patients [13,14]. Females, while also showing thalamic neuroinflammation, exhibited different patterns of regional involvement and signal characteristics. Advanced machine learning classification based on TSPO imaging features achieved 78% accuracy in discriminating chronic pain patients from healthy controls, with sex-specific feature importance emerging as a key finding [15]. Thalamic mean and maximum TSPO values were the most discriminating features in males, reflecting the intensity-based nature of their neuroinflammatory response. In contrast, higher-order statistical features such as kurtosis and entropy, which capture signal heterogeneity and spatial complexity, were more important classifiers in females, suggesting a more distributed or variable pattern of glial activation [15].

Single-cell RNA sequencing of spinal cord microglia following peripheral nerve injury has revealed fundamentally different microglial activation states between males and females at the molecular level. In males, nerve injury triggered the emergence of a distinct inflammatory microglial subtype characterized by robust upregulation of pro-inflammatory cytokines, including Il-1β, TNF, and IL6, alongside complement cascade components C1QA and C3 [22,48]. Notably, apolipoprotein E (Apo-E) emerged as the top differentially expressed gene in this male-specific microglial population, suggesting a role for lipid metabolism and cholesterol handling in male neuroinflammatory responses [22]. The temporal dynamics of this male-specific inflammatory microglial subtype are particularly striking: it first appeared at day 7 post-injury and persisted through chronic timepoints at day 28, indicating sustained pro-inflammatory signaling throughout the transition from acute to chronic pain [22]. In contrast, females showed entirely distinct microglial transcriptional states that were dominated by interferon-responsive genes and antigen presentation pathways rather than classical inflammatory cytokine production [22,49]. This divergence in microglial gene expression profiles between sexes suggests that males and females deploy fundamentally different innate immune programs in response to the same nerve injury stimulus, with males favoring a cytokine-driven inflammatory response and females engaging interferon and adaptive immune signaling pathways.

#### 3.4.2. Astrocyte Mechanisms: Emerging Sex Differences

Astrocyte activation patterns demonstrated region-specific and pain-type-specific sexual dimorphism, revealing that the role of astrocytes in chronic pain varies not only between sexes but also across different pain conditions and anatomical locations. In experimental osteoarthritis, females exhibited significantly higher GFAP-positive astrocyte density in the rostral ventromedial medulla (RVM), a critical brainstem region for descending pain modulation, compared to males (mean difference: 13.4 cells/mm^2^, 95% CI: 1.95–25.8, *p* = 0.025) [50]. Beyond simple numerical increases, these female astrocytes displayed enhanced mitotic activity and greater structural complexity as measured by Sholl analysis, indicating a more elaborate morphological transformation [50]. Remarkably, this pronounced female-specific RVM astrocyte activation was completely absent in males despite equivalent pain behaviors, suggesting that females and males achieve similar pain phenotypes through sexually dimorphic descending pain modulation circuits.

Conversely, in chronic neuropathic pain, the pattern reversed: males demonstrated higher overall GFAP expression across multiple brainstem regions, including the RVM and subnucleus reticularis dorsalis, with the notable exception of the area postrema, where females showed elevated GFAP [51]. This finding underscores that sex differences in astrocyte activation are not uniform but rather depend on the specific pain etiology and neuroanatomical context.

Translating these preclinical findings to human chronic pain, magnetic resonance spectroscopy studies in fibromyalgia patients (*n* = 43 patients, *n* = 16 controls) revealed elevated choline in the anterior insula—a putative neuroimaging marker of astrogliosis—that correlated significantly with pain interference (r = 0.41, *p* = 0.01) and reduced functional connectivity between anterior insula and putamen (r = −0.37, *p* = 0.03) [52]. The validity of choline as an astrogliosis biomarker was confirmed in a nonhuman primate neuroinflammation model, where cortical choline levels correlated with direct histological measurements of GFAP expression (Spearman r = 0.49, *p* = 0.03), providing critical cross-species validation for this non-invasive imaging approach [52].

#### 3.4.3. T Cell-Mediated Mechanisms: Female-Predominant Pathways

Multiple lines of evidence establish T cells, particularly CD4+ and CD8+ T cells, as critical mediators of chronic pain in females, representing a fundamentally different neuroimmune mechanism than the microglial pathways predominant in males [18,28,53]. Following peripheral nerve injury, females demonstrated substantially greater infiltration of T cells into both sciatic nerves and spinal cord compared to males, accompanied by distinct cytokine and chemokine profiles that favor adaptive immune cell recruitment [54]. Quantitative flow cytometry revealed this sex difference to be highly significant: CD3+ T cells comprised 8.2% of CD45+ immune cells in female spinal cord at day 7 post-injury compared to only 2.1% in males (*p* < 0.01), representing nearly a four-fold difference in T cell accumulation [54].

Beyond simple T cell infiltration, recent mechanistic studies have identified a novel female-specific pathway by which these T cells drive pain hypersensitivity. Pannexin-1 (Panx1) channels expressed on CD8+ T cells mediate the release of leptin specifically in females, with this adipokine acting as a potent pro-nociceptive mediator in the spinal cord microenvironment [28]. Nerve injury triggered coordinated increases in both spinal CD8+ T cell numbers and leptin levels in females but not males, establishing a sex-specific neuroimmune signaling axis [28].

The causal role of this CD8+ T cell-leptin pathway in female pain has been definitively established through multiple experimental approaches. Intrathecal administration of leptin-neutralizing antibody sex-specifically reversed mechanical allodynia in females, increasing paw withdrawal thresholds from 2.1 ± 0.4 g to 8.7 ± 1.2 g (*p* < 0.001), while having no effect in males [28]. Most compellingly, adoptive transfer experiments demonstrated that female-derived CD8+ T cells alone were sufficient to induce robust allodynia when transferred into naïve recipients, and this transferred pain phenotype was completely prevented by either leptin-neutralizing antibody or leptin siRNA knockdown [28]. These findings establish CD8+ T cell-derived leptin as a female-specific pain mediator and highlight the adaptive immune system as a therapeutic target in female chronic pain.

IL-16–CD4 signaling in CD3+ T cells emerged as another female-specific neuroimmune pathway driving chronic pain through distinct temporal dynamics and cellular mechanisms [55]. Following spinal nerve ligation, IL-16 mRNA expression in the spinal cord showed striking sex differences in its time course: females exhibited sustained elevation persisting beyond 28 days post-injury, whereas males demonstrated only transient IL-16 upregulation that resolved during the acute phase [55]. This prolonged IL-16 expression in females correlated with persistent pain behaviors and proved functionally critical, as both IL-16 knockdown and pharmacological CD4 receptor inhibition attenuated established mechanical allodynia in females only, leaving male pain responses unchanged [55].

Mechanistically, IL-16 induced activation of astrocytes but not microglia specifically in females, revealing a female-predominant T cell-to-astrocyte signaling cascade [55]. The T cell dependence of this astrocyte activation was confirmed by demonstrating that CD3 antibody treatment, which depletes T cells, completely suppressed IL-16-induced astrocyte activation, establishing that T cells serve as essential intermediaries between IL-16 signaling and astrocyte-mediated pain amplification in females [55].

These preclinical findings align with molecular profiling of human chronic pain patients. RNA sequencing of dorsal root ganglia from neuropathic pain patients (*n* = 50; 25 males, 25 females) revealed profound sex differences in immune gene expression that mirror the sexually dimorphic mechanisms identified in animal models [26]. Males showed increased expression of classical pro-inflammatory mediators, including IL1B, TNF, CXCL14, and oncostatin M (OSM), whereas females demonstrated elevated expression of chemokines CCL1 and CCL21, the opioid peptide PENK, and the pattern recognition receptor TLR3 [26].

Coexpression network analysis further delineated sex-specific molecular programs: JUN–FOS signaling, associated with immediate-early gene responses and neuronal plasticity, was enriched in males, while centromere protein genes were enriched in females, suggesting sex differences in cell cycle regulation or chromosomal organization [26]. Neuroimmune signaling pathways diverged fundamentally between sexes, with the oncostatin M–leukemia inhibitory factor–suppressor of cytokine signaling 1 (OSM–LIF–SOCS1) axis predominating in males, whereas the chemokine signaling cascade CCL1–CCL19–CCL21 was predominant in females [26]. These human data validate that the sex-specific neuroimmune mechanisms identified in preclinical models are conserved in human chronic pain pathophysiology.

#### 3.4.4. B-Cell Mechanisms: Predictors of Pain Chronification

Emerging evidence implicates B cells in the transition from acute to chronic pain, with sex- and age-specific effects that challenge traditional views of pain chronification as primarily a neuronal or microglial phenomenon [29,56]. In a prospective cohort study of adults presenting with acute low back pain (*n* = 108), baseline B cell maturation trajectories uniquely predicted which individuals would transition to chronic pain at follow-up, independent of self-reported pain intensity, sex, and age (OR = 2.8, 95% CI: 1.4–5.6, *p* = 0.004) [29]. This finding suggests that immune cell differentiation states at the time of initial injury contain prognostic information about pain chronification risk that is not captured by clinical pain ratings alone.

Flow cytometric immunophenotyping revealed the specific B cell alterations underlying this predictive relationship: individuals who subsequently transitioned to chronic pain exhibited altered B cell differentiation patterns at baseline, characterized by increased plasmablasts, antibody-secreting B cell precursors, and decreased naïve B cells compared to those who recovered [29]. This shift toward a more activated, antibody-producing B cell phenotype even during the acute pain phase suggests that humoral immune priming may facilitate pain chronification.

Sex-stratified analysis demonstrated that B cell inflammation markers were more strongly associated with chronic pain development in females, whereas monocyte-derived inflammatory markers predominated in males, reinforcing the theme of sexually dimorphic immune mechanisms in pain chronification [29]. Supporting the clinical relevance of B cells in female chronic pain, transcriptomic meta-analysis of circulating immune cells across six distinct chronic pain conditions (*n* = 142 patients, *n* = 154 controls) identified T-cell leukemia/lymphoma 1A (TCL1A), a gene critically involved in B cell function and implicated in autoimmunity, as differentially expressed specifically in females with chronic pain [57]. Moreover, TCL1A expression levels correlated significantly with neuropathic symptom severity (r = 0.48, *p* < 0.01), linking B cell molecular signatures to clinical pain phenotypes in female patients [57].

#### 3.4.5. Macrophage Mechanisms: Sex Differences in Polarization and Function

Macrophage contributions to chronic pain exhibited sex-specific polarization patterns and differential sensitivity to inflammatory stimuli, revealing that these peripheral immune cells play distinct roles in male versus female pain pathophysiology [58]. Females demonstrated markedly greater sensitivity to macrophage-derived mediators: small volumes of TNF-conditioned macrophage media were sufficient to induce robust mechanical hypersensitivity in females but failed to produce pain behaviors in males, indicating a lower threshold for macrophage-mediated pain signaling in females [58]. Paradoxically, despite this heightened initial sensitivity, TNF conditioning ultimately led to more rapid pain resolution in females compared to males, a phenomenon associated with altered immune cell recruitment profiles that may reflect enhanced pro-resolving immune responses [58].

At the cellular level, male and female macrophages exhibited intrinsic functional differences following TNF stimulation, including distinct cytokine secretion profiles and differential motility patterns, suggesting that sex chromosomes or prior hormonal exposure program macrophages to respond differently to identical inflammatory challenges [58]. These sex differences in macrophage biology have direct implications for pain chronification mechanisms.

The spatial organization of myeloid cell contributions to chronic pain also differs between sexes. Temporal analysis using myeloid-lineage TLR4 conditional knockout mice revealed that microglial Toll-like receptor 4 (TLR4), a pattern recognition receptor activated by damage-associated molecular patterns, contributes to early pain progression predominantly in males, with a significant but substantially less robust effect in females [59]. In contrast, females additionally relied on peripheral myeloid-lineage cells, particularly macrophages, or other TLR4-expressing cells outside the central nervous system to trigger chronic pain development [59]. This demonstrates not only temporal but also spatial divergence in immune mechanisms: males depend more heavily on central (spinal microglial) TLR4 signaling, whereas females engage both central and peripheral (macrophage) TLR4-dependent pathways, reflecting a more distributed immune contribution to pain chronification in females [59].

#### 3.4.6. Cytokine and Chemokine Profiles: Complex Sex-Specific Patterns

Meta-analysis of cytokine levels in fibromyalgia patients (*n* = 1772 participants across 22 studies) revealed significantly elevated circulating levels of multiple inflammatory mediators compared to healthy controls, including TNF (SMD = 0.36, 95% CI: 0.12–0.60, *p* = 0.003), IL-6 (SMD = 0.15, 95% CI: 0.003–0.29, *p* = 0.045), IL-8 (SMD = 0.26, 95% CI: 0.05–0.47, *p* = 0.01), and the anti-inflammatory cytokine IL-10 (SMD = 0.61, 95% CI: 0.34–0.89) [60]. However, substantial heterogeneity across studies (I^2^ = 39–71%) and inconsistent sex-stratified reporting severely limited the ability to interpret potential sex differences in these inflammatory profiles, representing a critical gap in the literature [60].

Individual studies with adequate sex stratification revealed nuanced patterns, though comprehensive sex-specific analyses remain limited. Serum neuropeptides, including corticotropin-releasing hormone, substance P, and hemokinin-1, along with inflammatory cytokines IL-6 and TNF, were elevated in fibromyalgia patients compared to controls, but sex-specific analyses were not reported despite the predominantly female patient population [61]. More sophisticated machine learning approaches integrating cytokine profiles with clinical phenotypes suggested that lowered IL-6 and IL-10 signaling, rather than elevated levels, may characterize fibromyalgia when controlling for important confounders, including sex, age, BMI, and psychiatric comorbidities, highlighting the complexity of interpreting peripheral cytokine data [62].

Cerebrospinal fluid (CSF) chemokine profiling provides more direct evidence of central nervous system neuroinflammation. In neuropathic pain patients (*n* = 27) versus controls (*n* = 11), CSF demonstrated elevated levels of multiple chemokines, including CXCL6, CXCL10, CCL8, CCL11, and CCL23, pointing to active neuroinflammatory processes within the central nervous system [63]. Notably, these same chemokines were also elevated in fibromyalgia CSF, suggesting shared neuroinflammatory mechanisms across distinct chronic pain conditions despite different peripheral etiologies [63].

Pain phenotype-specific analyses have revealed additional complexity in neuroimmune biomarkers. In chronic low back pain patients stratified by clinical phenotype (radicular versus axial pain), CSF cytokines, including CCL11, CD5, IL-8, and matrix metalloproteinase 10 (MMP-10), were associated in a nonlinear manner with back pain intensity specifically in radicular but not axial pain, suggesting that nerve root involvement engages distinct neuroinflammatory pathways [64]. However, these associations were not stratified by sex, representing another missed opportunity to examine sex-specific neuroimmune mechanisms in human chronic pain [64].

#### 3.4.7. BDNF: Sex-Dependent and Context-Dependent Effects

BDNF emerged as a sexually dimorphic neuromodulator with pro-nociceptive effects that operate predominantly in males through distinct molecular mechanisms [19,65]. In inflammatory pain models, BDNF drives pathological coupling with downregulation of the potassium-chloride cotransporter KCC2 in spinal dorsal horn neurons, disrupting inhibitory neurotransmission and promoting neuronal hyperexcitability, a process that occurs predominantly in males [19]. Direct experimental evidence for this sex difference came from intrathecal BDNF administration studies: exogenous BDNF induced robust mechanical allodynia in males but failed to produce pain hypersensitivity in females, demonstrating fundamental sex differences in neuronal responsiveness to this neurotrophin [19].

Hormonal manipulation experiments revealed that this sexual dimorphism is mediated by ovarian hormones rather than testicular androgens. Ovariectomy abolished the female resistance to BDNF-induced allodynia, rendering females as sensitive to BDNF as males and suggesting that estrogen or progesterone confer protective effects against BDNF-driven pain mechanisms [19]. Conversely, orchidectomy did not alter male BDNF sensitivity, indicating that the male phenotype operates through testosterone-independent mechanisms, likely reflecting organizational effects of early developmental androgen exposure or sex chromosome complement [19].

Translating these preclinical findings to human chronic pain has proven challenging, with human studies examining serum BDNF as a biomarker revealing context-dependent and sometimes contradictory associations. In a large cohort of older adults (*n* = 1932), higher serum BDNF was associated with increased odds of chronic pain (OR = 1.18 per SD increase, 95% CI: 1.04–1.34), with even stronger associations for severe pain (OR = 1.29, 95% CI: 1.07–1.56), though these relationships were modulated by sex and depression status in complex ways [66].

However, broader meta-analysis across diverse chronic pain conditions showed highly inconsistent findings that complicate interpretation. Some studies reported elevated BDNF, particularly in fibromyalgia (SMD 0.72, 95% CI: 0.12–1.31), while others showed no difference or even decreased levels compared to healthy controls [67,68]. This heterogeneity likely reflects differences in pain conditions studied, peripheral versus central BDNF measurement, timing of assessment, and unmeasured confounders. Critically, sex-stratified analyses were rarely reported in human BDNF studies, severely limiting the ability to determine whether the sexually dimorphic BDNF mechanisms identified in preclinical models are conserved in human chronic pain populations [67,68].

#### 3.4.8. Synthesis-Level Results, Bias, and Certainty of Evidence

Across individual syntheses, contributing studies varied in design, sample size, and methodological rigor. Syntheses of microglial and T cell–mediated mechanisms primarily included translational studies integrating preclinical models with human tissue, neuroimaging, or molecular data, generally exhibiting moderate risk of bias due to limited sample sizes and incomplete sex-stratified power. Cytokine and chemokine syntheses were dominated by observational human studies with heterogeneous assay methods and variable control of confounding, contributing to higher risk of bias and inconsistency. Imaging-based syntheses were based on smaller, well-characterized cohorts with lower risk of selection bias but limited generalizability. Across syntheses, the most common sources of bias included inadequate power for sex-specific analyses, incomplete reporting of hormonal status, and heterogeneity in outcome measurement. These factors were considered when interpreting the strength and consistency of findings within each synthesis.

Statistical syntheses were performed for outcomes with sufficient methodological and clinical comparability. Meta-analyses of inflammatory mediators in fibromyalgia demonstrated significantly higher circulating levels of multiple cytokines compared with healthy controls, with summary estimates reported as standardized mean differences and corresponding 95% confidence intervals, and moderate to substantial statistical heterogeneity as quantified by the I^2^ statistic. Synthesis of predictors of pain chronification yielded pooled odds ratios indicating increased risk associated with immune cell maturation profiles. Across quantitative syntheses, the direction of effect consistently indicated greater inflammatory or immune activation in chronic pain populations compared with controls. For other outcomes, including neuroimaging markers and mechanistic pathway activation, statistical synthesis was not feasible and results were synthesized narratively.

Formal statistical investigations of heterogeneity, including subgroup analyses and meta-regression, were not conducted due to insufficient numbers of studies with comparable sex-stratified outcomes and substantial clinical and methodological diversity. Qualitative exploration of heterogeneity across syntheses identified consistent patterns. Differences in pain condition, outcome definition, study design, and adequacy of sex-specific reporting contributed substantially to between-study variability. Syntheses involving neuroimaging and translational mechanistic studies tended to show greater consistency in effect direction, whereas cytokine and biomarker studies demonstrated greater heterogeneity related to assay methods and population characteristics. These observations informed interpretation of the synthesized findings.

Formal sensitivity analyses were not conducted due to the limited number of studies eligible for quantitative synthesis and substantial clinical and methodological heterogeneity. Qualitative assessment of robustness across syntheses demonstrated consistent direction of effects within major mechanistic domains, including male-predominant microglial pathways and female-predominant adaptive immune mechanisms. These patterns were observed across independent study designs, species, and measurement modalities, supporting the stability of the synthesized findings despite between-study variability.

Assessment of risk of bias due to missing results varied across syntheses. For quantitative syntheses including sufficient numbers of studies, visual inspection of funnel plots and Egger’s regression test did not demonstrate strong evidence of small-study effects, although statistical power to detect publication bias was limited. For most syntheses, formal assessment of reporting bias was not feasible due to the small number of contributing studies and substantial heterogeneity. Qualitative evaluation suggested a moderate risk of reporting bias, primarily related to selective reporting of sex-stratified outcomes and underreporting of null or negative findings. These considerations were taken into account when interpreting the synthesized results.

Certainty of evidence was assessed for each major outcome and synthesis using the GRADE framework. Overall certainty ranged from low to moderate across outcomes. Evidence supporting sex-specific microglial mechanisms in neuropathic pain and adaptive immune-mediated mechanisms in females was rated as moderate certainty, based on consistency of effect direction across independent studies and translational validation in human tissue, despite limitations related to sample size and study design. Evidence for cytokine and chemokine alterations in chronic pain was rated as low certainty due to substantial heterogeneity, risk of bias, and inconsistent sex-stratified reporting. Evidence for predictors of pain chronification involving immune cell profiles was rated as low to moderate certainty. Certainty was commonly downgraded for risk of bias, inconsistency, indirectness, and imprecision.

### 3.5. Neuroimmune–Endocrine Interactions: Hormonal Modulation of Immune Mechanisms

Emerging evidence demonstrates that sex hormones directly modulate neuroimmune mechanisms, providing mechanistic insight into sex differences [69,70]. Estrogen receptor signaling in microglia and macrophages influences their activation states, with 17β-estradiol generally exerting anti-inflammatory effects through ERα and ERβ pathways [69,71,72]. However, the relationship is complex and context-dependent: physiological estrogen fluctuations across the menstrual cycle modulate pain sensitivity and immune responses, with some studies showing pro-nociceptive effects during specific cycle phases [73,74].

Testosterone also modulates neuroimmune function, with generally anti-inflammatory and analgesic effects [75,76]. Gonadectomy studies in rodents revealed that testosterone depletion enhanced microglial activation and pain behaviors in males, effects reversed by testosterone replacement [75,77,78]. Conversely, in females, ovariectomy sometimes enhanced microglial-dependent pain mechanisms, suggesting that ovarian hormones normally suppress microglial contributions [74].

Progesterone and its neurosteroid metabolites (particularly allopregnanolone) emerged as potent modulators of neuroinflammation and pain [79,80]. Progesterone treatment reduced microglial activation, pro-inflammatory cytokine production, and pain behaviors in both sexes, but with greater efficacy in females [81,82]. Mechanistically, progesterone acts through classical progesterone receptors and through conversion to allopregnanolone, which modulates gamma-aminobutyric acid type A (GABA-A) receptors and reduces neuroinflammation [83,84].

Prolactin represents another sexually dimorphic neuroimmune modulator [27,85]. Elevated prolactin levels in chronic pain patients correlated with pain severity and fatigue, particularly in females [86]. Mechanistically, prolactin enhances T cell and B cell function, promotes pro-inflammatory cytokine production, and may contribute to the female predominance of autoimmune-associated pain conditions [20,85,87].

### 3.6. Sex Chromosome Effects Independent of Hormones

Beyond hormonal mechanisms, sex chromosome complement (XX vs. XY) directly influences neuroimmune function independent of gonadal hormones [88,89]. Studies using the “four core genotypes” mouse model, which dissociates gonadal sex from sex chromosome complement, revealed that XX mice exhibited enhanced microglial reactivity and inflammatory responses compared to XY mice, regardless of gonadal phenotype [89,90].

X-chromosome genes implicated in sexually dimorphic neuroimmune function include Toll-like receptor 7 (TLR7) (which escapes X-inactivation in females, leading to higher expression and enhanced innate immune responses) and genes encoding microRNAs that regulate inflammatory pathways [91,92]. Y-chromosome genes, particularly sex-determining region Y (SRY) (which is expressed in brain microglia and neurons beyond its developmental role), may also contribute to male-specific neuroimmune phenotypes [93].

### 3.7. Age–Sex Interactions in Neuroimmune Pain Mechanisms

The intersection of age and sex profoundly influences neuroimmune pain mechanisms, with distinct patterns across the lifespan [94,95]. In younger adults, sex differences in microglial mechanisms are most pronounced, whereas in older adults, adaptive immune mechanisms (particularly T and B cells) show greater sexual dimorphism [90].

Menopause represents a critical transition point where declining estrogen levels alter neuroimmune function [96,97]. Postmenopausal women exhibit enhanced microglial reactivity, elevated pro-inflammatory cytokines, and increased chronic pain prevalence compared to premenopausal women [98,99]. Hormone replacement therapy partially reverses these changes, reducing neuroinflammation and pain in some but not all women, suggesting individual variability in hormone–immune interactions [94,96].

In males, age-related testosterone decline (andropause) similarly influences neuroimmune function, with older men showing enhanced inflammatory responses and altered pain processing [94]. However, the magnitude of age-related changes appears smaller in males than in females, potentially contributing to the female predominance of chronic pain in older adults [97].

### 3.8. Comorbidity-Specific Neuroimmune Mechanisms

Sex differences in neuroimmune pain mechanisms vary across comorbid conditions, particularly psychiatric and metabolic disorders. In patients with comorbid chronic pain and depression, females exhibited higher levels of pro-inflammatory cytokines (IL-6, TNF, C-reactive protein (CRP) compared to males with the same comorbidity profile [100,101]. This enhanced inflammation in females with pain-depression comorbidity may reflect bidirectional neuroimmune-psychiatric interactions mediated by shared pathways, including kynurenine metabolism and microglial activation [102,103,104].

Obesity and metabolic syndrome amplify sex differences in neuroimmune pain mechanisms [11,105]. Adipose tissue-derived inflammatory mediators (adipokines, cytokines) interact with sex hormones to modulate central and peripheral neuroimmune function [106,107]. In obese females, elevated leptin levels enhance T cell-mediated pain mechanisms, whereas in obese males, adipose tissue macrophage activation predominates [28,105]. These sex-specific obesity-pain-immune interactions have important implications for pain management in the context of the obesity epidemic [108].

### 3.9. Genetic and Epigenetic Regulation of Sex Differences

Emerging genomic and epigenomic studies reveal sex-specific genetic and epigenetic regulation of neuroimmune pain pathways. Genome-wide association studies of chronic pain identified sex-specific genetic variants, with some loci showing significant genotype-by-sex interactions [6,109]. Notably, variants in immune-related genes (human leukocyte antigen [HLA] region, cytokine genes, chemokine receptors) demonstrated stronger associations with chronic pain in females than males [6,110].

Epigenetic mechanisms, particularly deoxyribonucleic acid (DNA) methylation and histone modifications, exhibit sexual dimorphism in pain-related genes [111,112]. In dorsal root ganglia from chronic pain patients, sex-specific DNA methylation patterns were identified in genes encoding ion channels, neurotransmitter receptors, and immune mediators [113,114]. These epigenetic differences may arise from sex hormone effects, sex chromosome dosage, or developmental programming, and contribute to persistent sex differences in pain vulnerability [115,116].

MicroRNAs represent another layer of sex-specific gene regulation in neuroimmune pain mechanisms [117,118]. Several microRNAs show sexually dimorphic expression in pain-relevant tissues, with some (e.g., miR-let-7, miR-223) regulating microglial activation and inflammatory responses in a sex-dependent manner [119,120]. Therapeutic targeting of sex-specific microRNAs represents a potential precision medicine approach for chronic pain [116].

### 3.10. Pharmacological Implications: Sex Differences in Treatment Response

The sexually dimorphic neuroimmune mechanisms have profound implications for pain pharmacotherapy, with emerging evidence of sex-specific drug efficacy. Opioid analgesics demonstrate well-established sex differences, with females generally requiring lower doses for equivalent analgesia but experiencing more adverse effects [121,122,123]. Mechanistically, sex differences in μ-opioid receptor expression, signaling, and neuroimmune interactions (particularly opioid-induced microglial activation) contribute to these pharmacological disparities [51,124,125].

Non-opioid analgesics also exhibit sexual dimorphism in efficacy and mechanisms. Nonsteroidal anti-inflammatory drugs (NSAIDs) and cyclooxygenase-2 (COX-2) inhibitors show greater efficacy in males in some pain models, potentially reflecting male-predominant prostaglandin-mediated mechanisms [126,127]. Conversely, drugs targeting T cell or B cell function demonstrate greater efficacy in females in preclinical models, consistent with female-predominant adaptive immune mechanisms [53,128].

Emerging immunomodulatory therapies for chronic pain must account for sex differences in target engagement and efficacy [129,130]. Microglial inhibitors show male-biased efficacy in some neuropathic pain models [43,131,132], whereas therapies targeting T cells or cytokines may be more effective in females [28,95]. Clinical trials of neuroimmune-targeted pain therapies have rarely stratified by sex or tested for sex-by-treatment interactions, representing a critical knowledge gap [130].

### 3.11. Precision Medicine Approaches: Toward Sex-Specific Pain Management

The comprehensive evidence for sexually dimorphic neuroimmune pain mechanisms supports precision medicine approaches that incorporate biological sex as a key stratification variable. Proposed frameworks include sex-stratified diagnostic algorithms incorporating neuroimmune biomarkers, sex-specific treatment algorithms prioritizing mechanism-matched therapies, and integration of hormonal status, age, and comorbidities into sex-based treatment selection.

Biomarker-guided therapy represents a particularly promising precision medicine approach. Patients could undergo baseline assessment of neuroimmune mechanisms to identify dominant pathways, with treatment selection based on individual mechanism profiles rather than sex alone. This approach acknowledges that while population-level sex differences exist, individual patients may exhibit mechanisms atypical for their sex.

### 3.12. Heterogeneity and Limitations

Several sources of heterogeneity and methodological limitations warrant consideration when interpreting these findings. First, substantial heterogeneity exists within each sex, with some females exhibiting “male-typical” mechanisms and vice versa, emphasizing that sex differences represent population-level trends rather than absolute dichotomies. Second, most studies examined binary sex (male/female) without considering gender identity, transgender individuals, or intersex conditions, limiting generalizability.

Third, preclinical-to-clinical translation challenges are substantial. Many sex differences observed in rodent models have not been validated in humans, and species differences in immune system organization may limit translatability. Fourth, confounding by sex-associated variables (hormonal status, medication use, comorbidities, psychosocial factors) complicates interpretation of observational human studies.

Fifth, publication bias may inflate apparent sex differences, as studies finding no sex differences may be less likely to be published. Finally, the field has historically focused on specific mechanisms (microglia, T cells) while potentially overlooking other sexually dimorphic pathways (e.g., mast cells, neutrophils, complement), representing a knowledge gap [18,130,133].

### 3.13. Summary of Key Findings

This systematic review identified robust, convergent evidence for sexually dimorphic neuroimmune mechanisms in chronic pain across multiple levels of analysis:

(1) Microglial P2X4R signaling represents a male-predominant mechanism in neuropathic pain, validated across preclinical models, human tissue, and neuroimaging studies.
(2) T cell-mediated mechanisms, particularly CD4+ and CD8+ T cells signaling through IL-16, leptin, and chemokine pathways, drive chronic pain predominantly in females.
(3) B cell maturation trajectories predict transition from acute to chronic pain in a sex- and age-specific manner, with stronger associations in females.
(4) Astrocyte activation exhibits region-specific and pain-type-specific sexual dimorphism, with female-predominant activation in descending modulatory circuits.
(5) Cytokine and chemokine profiles differ between sexes, though substantial heterogeneity and methodological limitations complicate interpretation.
(6) BDNF demonstrates male-predominant pro-nociceptive effects in preclinical models, with complex and context-dependent associations in humans.
(7) Sex hormones, sex chromosomes, age, comorbidities, and genetic/epigenetic factors interact to produce the observed sexual dimorphism in neuroimmune pain mechanisms.
(8) Pharmacological responses to analgesics and immunomodulatory therapies exhibit sex-specific patterns, supporting precision medicine approaches.

These findings establish that chronic pain is not a single disease but rather a collection of mechanistically distinct conditions that differ fundamentally between the sexes, necessitating sex-specific diagnostic and therapeutic approaches

## 4. Discussion

### 4.1. Principal Findings and Clinical Implications

This systematic review synthesized evidence from studies demonstrating that sexually dimorphic neuroimmune mechanisms fundamentally shape chronic pain pathophysiology, with profound implications for precision medicine approaches to pain management [4,18,20]. The convergent findings across preclinical models, human tissue studies, neuroimaging investigations, and clinical cohorts establish that chronic pain is not a unitary condition but rather represents mechanistically distinct syndromes that differ between the sexes at molecular, cellular, and systems levels [19,28].

The most robust and clinically actionable finding is the male-predominant role of microglial P2X4R signaling versus female-predominant T cell-mediated mechanisms in neuropathic pain [28,42,43,53]. This fundamental divergence in immune cell involvement has been validated across multiple pain models, anatomical sites, and species, including humans, suggesting it represents a conserved evolutionary feature rather than an experimental artifact [46,48]. The demonstration that prolactin selectively sensitizes female nociceptors while orexin B selectively sensitizes male nociceptors, conserved from mice through non-human primates to humans, provides compelling evidence for qualitatively distinct pain mechanisms that could be therapeutically targeted [20,27,85].

### 4.2. Translational Challenges and Opportunities

Despite robust preclinical evidence for sexually dimorphic pain mechanisms, translation to clinical practice faces substantial challenges [134,135]. First, most existing pain therapies were developed and tested predominantly in male animals and subsequently in clinical trials that either excluded women or failed to analyze sex-specific efficacy [4,18]. This historical bias may partially explain the high failure rate of novel analgesics in clinical development, as drugs targeting male-predominant mechanisms (e.g., microglial inhibitors) may show efficacy in male-dominated preclinical studies but fail in patient populations that are predominantly female [136,137,138].

Second, species differences in immune system organization and pain processing complicate direct translation [139,140,141]. While key findings such as BDNF-mediated sexual dimorphism in spinal hyperexcitability have been validated in human tissue [19], many mechanistic insights derive exclusively from rodent models. Rodents and humans differ in immune cell composition, hormone cycling patterns, and pain behavior assessment, necessitating cautious interpretation of preclinical findings [134,142].

Third, individual variability within each sex is substantial, with some females exhibiting “male-typical” mechanisms and vice versa. This heterogeneity suggests that while population-level sex differences exist, individual patients may require mechanism-based rather than sex-based treatment selection [20]. Biomarker-guided approaches that identify dominant neuroimmune pathways in individual patients, regardless of sex, may prove more effective than blanket sex-stratified treatment algorithms [143,144].

### 4.3. Precision Medicine Framework for Sex-Specific Pain Management

The evidence supports a multi-tiered precision medicine framework that incorporates biological sex as a key stratification variable while acknowledging individual mechanistic heterogeneity [1,143]. This framework would include: (1) sex-stratified diagnostic algorithms incorporating neuroimmune biomarkers such as TSPO-PET imaging for microglial activation, peripheral immune cell phenotyping, and cytokine profiling [13,29]; (2) mechanism-matched therapy selection prioritizing microglial inhibitors or P2X4R antagonists for patients with male-typical mechanisms and T cell-targeted therapies or leptin-neutralizing approaches for those with female-typical mechanisms [28,43]; and (3) integration of hormonal status, age, comorbidities, and genetic/epigenetic profiles to refine treatment selection beyond binary sex categories [145,146].

Emerging therapeutic targets with sex-specific potential include prolactin receptor antagonists for female-predominant pain and orexin receptor modulators for male-predominant pain, both validated in human sensory neurons [20,85,86]. Additionally, pannexin-1 channel blockers show promise for both sexes but through divergent mechanisms (microglial VEGF-A in males, T cell leptin in females), suggesting that combination approaches or patient stratification may optimize efficacy [28].

### 4.4. Clinical Trial Design Considerations

The sexually dimorphic mechanisms identified in this review have critical implications for clinical trial design [4,147]. Current pain clinical trials often enroll unbalanced sex ratios and rarely conduct pre-specified sex-stratified analyses, potentially masking sex-specific efficacy signals [5,148]. Future trials should: (1) ensure adequate representation of both sexes with sufficient power for sex-stratified analyses [147,149]; (2) collect and report hormonal status (menstrual cycle phase, menopausal status, hormone therapy use) as these factors modulate neuroimmune mechanisms [94,95]; (3) incorporate mechanism-based biomarkers to identify patients most likely to respond based on their dominant neuroimmune pathways rather than sex alone [145,150]; and (4) consider re-analysis of failed trials with sex-stratified subgroup analyses, as therapies that failed in mixed populations may show efficacy in one sex [20,151].

It is critical that future intervention trials are adequately powered to detect sex-based heterogeneity in treatment effects. This involves not only ensuring sufficient sample sizes for male and female participants but also incorporating analyses that control for sex as a factor influencing treatment response. By doing so, these studies can better identify potential sex-based moderation of treatment effects, allowing for more personalized and effective therapeutic strategies.

### 4.5. Knowledge Gaps and Future Research Directions

Despite substantial progress, critical knowledge gaps remain. First, most mechanistic studies have focused on neuropathic pain models, with less comprehensive investigation of sex differences in inflammatory, visceral, cancer-related, and centralized pain conditions [53,95]. Emerging evidence suggests that sexually dimorphic mechanisms may vary across pain types, necessitating condition-specific investigation [152].

Second, the role of adaptive immune cells beyond T cells, particularly B cells, regulatory T cells, and innate lymphoid cells, remains incompletely characterized [29,56,153]. Recent findings that B cell maturation trajectories predict chronic pain development in a sex- and age-specific manner highlight the need for comprehensive immune profiling across the pain chronicity spectrum [29,154,155,156].

Third, neuroimmune–endocrine interactions require deeper mechanistic investigation [7,157]. While sex hormones clearly modulate neuroimmune function [69,70], the precise molecular mechanisms, including receptor-mediated signaling, epigenetic programming, and metabolic regulation, remain incompletely understood. The observation that ovariectomy can masculinize pain mechanisms in females suggests that hormonal interventions might modulate pain vulnerability, but clinical translation requires careful investigation [19].

Fourth, sex chromosome effects independent of gonadal hormones warrant further study [88,89]. Evidence from four core genotypes models demonstrates that XX versus XY chromosome complement influences neuroimmune function independently of gonadal sex, but the specific genes and mechanisms involved remain largely unknown [90]. X-linked genes that escape inactivation (e.g., TLR7) and Y-chromosome genes expressed in brain (e.g., SRY) represent promising targets for investigation [158,159].

Fifth, age–sex interactions across the lifespan require systematic investigation [94,97,160]. Sex differences in pain mechanisms appear to vary across developmental stages, reproductive transitions (puberty, pregnancy, menopause, andropause), and aging, but comprehensive longitudinal studies are lacking [96]. Understanding how neuroimmune mechanisms evolve across the lifespan in each sex could inform age- and sex-specific prevention and treatment strategies.

Sixth, the intersection of sex with other biological and social determinants of pain—including race/ethnicity, socioeconomic status, trauma exposure, and comorbid conditions—remains underexplored. Pain is a biopsychosocial phenomenon [1,161,162], and sex differences in pain likely reflect complex interactions between biological mechanisms and social/environmental factors that shape pain experience and treatment access [148].

Seventh, pharmacological sex differences beyond opioids require comprehensive investigation [121,125,163]. While opioid sex differences are well-documented, systematic evaluation of sex-specific efficacy and mechanisms for non-opioid analgesics, immunomodulatory therapies, and emerging treatments (e.g., cannabinoids, ketamine, neuromodulation) is needed [164].

### 4.6. Methodological Considerations and Limitations

Several methodological limitations warrant consideration when interpreting this review’s findings. First, publication bias may inflate apparent sex differences, as studies finding no sex differences may be less likely to be published or prominently featured. This bias could lead to overestimation of the magnitude and consistency of sexually dimorphic mechanisms.

Second, most studies examined binary biological sex (male/female) without considering gender identity, transgender individuals, or intersex conditions [4,147]. Gender, the social and psychological aspects of being male or female, influences pain experience, reporting, and treatment-seeking behavior, but few studies distinguish biological sex from gender [165]. Additionally, transgender individuals receiving hormone therapy represent an important population for understanding hormone–pain relationships but are rarely included in research [166].

Third, confounding by sex-associated variables complicates interpretation of observational human studies. Females and males differ in numerous factors beyond neuroimmune mechanisms, including pain catastrophizing, coping strategies, healthcare utilization, medication adherence, and comorbid conditions, that could contribute to observed sex differences in pain outcomes. Disentangling biological mechanisms from psychosocial and behavioral factors requires carefully designed studies with comprehensive covariate assessment.

Fourth, heterogeneity in pain assessment methods, immune assays, and study populations limits cross-study comparability. Cytokine studies, in particular, showed substantial heterogeneity in findings, likely reflecting differences in sample types (serum, plasma, CSF), assay methods, patient populations, and timing of assessment relative to pain onset.

Fifth, most preclinical studies used young adult animals, limiting generalizability to pediatric and geriatric populations. Age-related changes in immune function, hormone levels, and pain processing may alter sexually dimorphic mechanisms, necessitating age-specific investigation [94].

The correlations in our analysis were statistically significant, but the variability explained was modest, with a maximum of 25%, indicating other factors may contribute to the observed outcomes. Similarly, the small to moderate SMDs reflect modest effect sizes, which may be influenced by factors like within-sex heterogeneity discussed in Section 3.12. Further research is needed to explore additional contributors to the variability, including unmeasured confounders and individual biological differences.

### 4.7. Implications for Drug Development

The sexually dimorphic mechanisms identified in this review have profound implications for analgesic drug development [130,136,137]. Traditional drug development paradigms that test compounds in male animals and subsequently in mixed-sex clinical populations may systematically miss sex-specific therapeutic opportunities and fail to identify sex-specific safety concerns [18].

A revised drug development paradigm should include: (1) mandatory inclusion of both sexes in preclinical efficacy and safety studies with adequate power for sex-stratified analyses [147]; (2) mechanistic investigation of sex-specific target engagement and downstream signaling; (3) early-phase clinical trials designed to detect sex differences in pharmacokinetics, pharmacodynamics, efficacy, and adverse effects; and (4) development of companion diagnostics to identify patients most likely to benefit from sex-specific or mechanism-specific therapies.

Promising sex-specific therapeutic targets emerging from this review include: (1) P2X4R antagonists for male-predominant microglial pain mechanisms [42,43]; (2) T cell-targeted therapies (e.g., fingolimod, anti-CD4/CD8 antibodies) for female-predominant adaptive immune mechanisms [28,167]; (3) leptin-neutralizing antibodies or leptin receptor antagonists for female-predominant leptin-mediated pain [28]; (4) prolactin receptor antagonists for female-selective nociceptor sensitization [20,27,85]; (5) orexin receptor modulators for male-selective nociceptor sensitization [20]; and (6) pannexin-1 channel blockers with potential efficacy in both sexes through divergent mechanisms [28].

### 4.8. Broader Implications for Pain Medicine

Beyond drug development, the findings of this review have broader implications for pain medicine practice and policy. First, pain assessment tools and diagnostic criteria may need sex-specific calibration, as males and females may express and report pain differently due to both biological and sociocultural factors [168]. Second, clinical practice guidelines should incorporate sex-specific recommendations where evidence supports differential treatment approaches [1]. Third, pain education for healthcare providers should emphasize sex and gender differences to reduce bias in pain assessment and treatment [4,97].

Fourth, health policy and insurance coverage decisions should consider sex-specific treatment needs [165]. If certain therapies prove more effective in one sex, coverage policies should ensure equitable access to sex-appropriate treatments. Fifth, patient education materials should acknowledge sex differences in pain mechanisms and treatment responses, empowering patients to engage in shared decision-making about sex-informed treatment options.

### 4.9. Integration with Existing Pain Frameworks

The sexually dimorphic neuroimmune mechanisms identified in this review integrate with and extend existing pain frameworks. The biopsychosocial model of pain—which emphasizes the interaction of biological, psychological, and social factors—is enriched by recognition that biological mechanisms themselves are sexually dimorphic and interact with sex-specific psychosocial factors [1,161,162]. The concept of pain as a “disease” rather than merely a symptom is strengthened by evidence that distinct pathophysiological mechanisms drive pain in different patient subgroups defined by sex and other characteristics.

The central sensitization framework, which posits that chronic pain reflects maladaptive plasticity in central pain processing, is refined by evidence that the cellular and molecular mechanisms of central sensitization differ between sexes [19,169]. Male-predominant BDNF–KCC2–NMDAR coupling versus female-predominant mechanisms (which remain incompletely characterized) suggest that “central sensitization” is not a unitary phenomenon but rather encompasses sex-specific pathways [19,27].

### 4.10. Evolutionary and Comparative Perspectives

The evolutionary origins and adaptive significance of sexually dimorphic pain mechanisms remain speculative but merit consideration. Sexual dimorphism in immune function is widespread across species and likely reflects differential selective pressures related to reproduction, pathogen exposure, and life history strategies [53]. Females generally mount stronger immune responses than males, conferring enhanced pathogen resistance but increased autoimmune disease risk [130]. The female-predominant role of adaptive immunity in chronic pain may represent a maladaptive consequence of this generally adaptive immune enhancement.

The male-predominant role of microglia may reflect testosterone-mediated immune suppression that reduces adaptive immune contributions while preserving innate immune surveillance. Alternatively, sex differences in pain mechanisms may reflect differential reproductive costs of pain-related disability, with females requiring more robust pain responses to protect offspring during vulnerable periods. Comparative studies across species with varying reproductive strategies could illuminate the evolutionary origins of sexually dimorphic pain mechanisms [18,95].

### 4.11. Ethical Considerations

The recognition of sexually dimorphic pain mechanisms raises important ethical considerations [147,170]. First, historical exclusion of females from pain research represents a significant ethical failure that has resulted in knowledge gaps and potentially suboptimal treatment for female patients [4,18]. Rectifying this requires not only inclusion of females in future research but also targeted investigation of female-predominant mechanisms that have been neglected.

Second, sex-specific treatment recommendations must be implemented carefully to avoid reinforcing harmful gender stereotypes or biases. Healthcare providers must distinguish biological sex differences from gender-based assumptions about pain tolerance, treatment-seeking behavior, or medication preferences. Third, transgender and gender-diverse individuals require specific consideration, as their pain mechanisms may reflect complex interactions between chromosomal sex, gonadal hormones (endogenous and exogenous), and gender identity [166].

Fourth, equitable access to sex-specific therapies must be ensured, avoiding scenarios where effective treatments are available only to one sex due to research priorities or coverage decisions [165,168]. Fifth, patient autonomy in treatment selection must be preserved, with sex-informed recommendations presented as evidence-based guidance rather than prescriptive mandates.

## 5. Conclusions

This systematic review demonstrates that chronic pain is fundamentally shaped by sexually dimorphic neuroimmune mechanisms that operate across peripheral, spinal, and supraspinal levels. Convergent human and translational evidence indicates that male-predominant microglial pathways, particularly P2X4R–BDNF-dependent signaling, contrast with female-predominant adaptive immune mechanisms driven by T cells, B cells, and cytokine-mediated communication with astrocytes and nociceptors. These differences are further modulated by sex hormones, sex chromosome complement, age, comorbidities, and genetic and epigenetic regulation, producing distinct biological trajectories of pain chronification in males and females.

The findings challenge the long-standing assumption that chronic pain mechanisms are sex-neutral and highlight limitations of therapeutic strategies developed without consideration of sex as a biological variable. While heterogeneity within each sex underscores the need for mechanism-based rather than purely sex-based treatment selection, biological sex remains a major determinant of dominant neuroimmune pathways. Future research must prioritize adequately powered sex-stratified analyses, integration of neuroimmune biomarkers, and clinical trial designs capable of detecting sex-by-treatment interactions. Incorporating sex-informed neuroimmune mechanisms into pain research and clinical practice is essential for advancing precision medicine and improving outcomes for individuals living with chronic pain.

## Figures and Tables

**Table 1:** Medical databases and search strategies used for the systematic review.

Database	Search Strategy	Results
PubMed (MEDLINE)	(“Chronic Pain”[MeSH] OR “Pain, Chronic”[MeSH] OR chronic pain[tiab] OR neuropathic pain[tiab] OR inflammatory pain[tiab] OR fibromyalgia[tiab] OR osteoarthritis[tiab] OR migraine[tiab] OR chronic low back pain[tiab]) AND (“Neuroinflammation”[MeSH] OR neuroinflammation[tiab] OR neuroimmune[tiab] OR “Microglia”[MeSH] OR microglia[tiab] OR “Astrocytes”[MeSH] OR astrocyte*[tiab] OR cytokine*[tiab] OR chemokine*[tiab] OR immune cell*[tiab] OR glial cell*[tiab]) AND (“Sex Characteristics”[MeSH] OR sex difference*[tiab] OR sex-specific[tiab] OR gender difference*[tiab] OR male[tiab] OR female[tiab])	1523
Embase (Emtree)	(‘chronic pain’/exp OR ‘neuropathic pain’/exp OR ‘inflammatory pain’/exp OR fibromyalgia/exp OR osteoarthritis/exp OR migraine/exp OR ‘chronic low back pain’/exp) AND (‘neuroinflammation’/exp OR neuroimmune:ti,ab OR ‘microglia’/exp OR microglia:ti,ab OR ‘astrocyte’/exp OR astrocyte*:ti,ab OR cytokine*:ti,ab OR chemokine*:ti,ab OR ‘immune cell’/exp OR ‘glial cell’/exp) AND (‘sex difference’/exp OR ‘sexual dimorphism’/exp OR sex-specific:ti,ab OR male:ti,ab OR female:ti,ab)	1687
Web of Science	TS=((“chronic pain” OR neuropathic pain OR inflammatory pain OR fibromyalgia OR osteoarthritis OR migraine OR “chronic low back pain”) AND (neuroinflammation OR neuroimmune OR microglia OR astrocyte* OR cytokine* OR chemokine* OR immune cell* OR glial cell*) AND (“sex difference*” OR “sexual dimorphism” OR sex-specific OR male OR female))	982
Cochrane CENTRAL	(chronic pain OR neuropathic pain OR inflammatory pain OR fibromyalgia OR osteoarthritis OR migraine OR chronic low back pain) AND (neuroinflammation OR neuroimmune OR microglia OR astrocyte* OR cytokine* OR chemokine* OR immune cell* OR glial cell*) AND (sex difference* OR sexual dimorphism OR sex-specific OR male OR female)	398
PsycINFO	(DE “Chronic Pain” OR chronic pain OR neuropathic pain OR fibromyalgia OR migraine) AND (neuroinflammation OR neuroimmune OR microglia OR astrocyte* OR cytokine* OR immune*) AND (sex differences OR sexual dimorphism OR sex-specific OR male OR female)	157
CINAHL	(MH “Chronic Pain+” OR chronic pain OR neuropathic pain OR fibromyalgia OR migraine) AND (MH “Neuroinflammation” OR neuroimmune OR microglia OR astrocyte* OR cytokine* OR immune*) AND (sex differences OR sexual dimorphism OR sex-specific OR male OR female)	100
ClinicalTrials.gov	Condition/Disease: chronic pain; Other terms: neuroinflammation OR microglia OR immune OR sex differences; Study type: all	43

The asterisk (*) serves as a wildcard or truncation symbol in keyword searches. Its primary function is to expand a search term by finding all words that begin with the letters you specify.

## Data Availability

Template data collection forms, extracted study data and analytic datasets generated during the current review are not publicly available. These materials were derived from published studies and were used solely for the purposes of this systematic review. Analytic code was not generated, as quantitative syntheses were conducted using standard meta-analytic procedures. Additional materials related to the review are available from the corresponding author upon reasonable request.
